# Neuroform atlas stent-assisted coiling of tiny wide-necked intracranial aneurysms

**DOI:** 10.3389/fneur.2022.1020785

**Published:** 2022-11-10

**Authors:** Linggen Dong, Xiheng Chen, Jiejun Wang, Longhui Zhang, Zhiqiang Zhao, Qichen Peng, Peng Liu, Ming Lv

**Affiliations:** ^1^Department of Neurointervention, Beijing Tiantan Hospital, Capital Medical University, Beijing, China; ^2^Beijing Neurosurgical Institute, Beijing Tiantan Hospital, Capital Medical University, Beijing, China; ^3^Department of Neurosurgery, Beijing Tiantan Hospital, Capital Medical University, Beijing, China

**Keywords:** Neuroform atlas stent, tiny intracranial aneurysms, wide-necked, stent-assisted coiling, endovascular treatment

## Abstract

**Objective:**

To investigate the safety and efficacy of Neuroform Atlas stent-assisted coiling for the treatment of tiny wide-necked intracranial aneurysms and evaluate risk factors associated with procedure-related complications.

**Methods:**

We retrospectively examined 46 patients with 46 tiny wide-necked aneurysms who were treated using Atlas stent-assisted coiling at our institution from August 2020 to May 2022. Patient and aneurysm characteristics, procedural details, procedure-related complications, and angiographic and clinical outcomes were analyzed.

**Results:**

A total of 10 patients presented with aneurysmal rupture. Atlas stent placement was successful in all patients. Angiography immediately after the procedure showed complete occlusion in 38 patients (82.6%), neck remnant in 7 (15.2%), and partial occlusion in 1 (2.2%). The mean angiographic follow-up was 8.4 months (range, 6–16). At the last follow-up, angiography showed complete occlusion in 41 patients (89.1%) and neck remnant in 5 (10.9%). No aneurysm recurrence or in-stent stenosis occurred. Incidence of procedure-related complications was 10.8% (intraprocedural aneurysm rupture, two cases; acute thrombosis, two cases; and coil migration, one case); only one patient (2.2%) experienced procedural neurological morbidity. The mean clinical follow-up was 9.7 months. A favorable outcome was achieved in 45 patients (97.8%). In univariate logistic regression analysis, aneurysm size (odds ratio, 4.538; *P* = 0.045) was significantly associated with procedure-related complications. However, multivariate analysis found no independent risk factors.

**Conclusion:**

Atlas stent-assisted coiling of tiny wide-necked intracranial aneurysms is feasible and effective. Outcomes and occlusion rates are favorable and morbidity is low. The complication rate may be higher in larger tiny aneurysms.

## Introduction

Aneurysms with a maximum diameter of ≤ 3 mm are defined as tiny (1–3). A greater number of tiny aneurysms are being diagnosed owing to recent advances in neuroradiological techniques and improvements in imaging resolution (4). Treatment of these aneurysms is particularly challenging because they are characterized by thin fragile walls. When surgically clipping a tiny aneurysm, partial clipping of the parent artery is frequently required to prevent the clip from falling off (5). When performing endovascular embolization, coil stabilization within the sac is difficult, particularly in wide-necked aneurysms; in addition, subarachnoid hemorrhage (SAH) may result from a catheter or coil piercing the fragile aneurysm wall (6).

Management of tiny wide-necked intracranial aneurysms is controversial. Reported rupture rates are low, ranging from 0 to 0.4% (7). However, tiny aneurysms account for 15% of all ruptured aneurysms, which suggests that rupture of tiny aneurysms is not uncommon and that they should be treated (8).

In May 2020, the Neuroform Atlas stent (Stryker Neurovascular, Fremont, CA, USA) was introduced in China. Since then, it has been widely used to treat wide-necked aneurysms. The Atlas stent is a self-expanding laser-cut nitinol stent that is delivered using a low-profile microcatheter (size, 0.0165–0.017 inch). Its novel mixed open-cell/closed-cell design enhances stability within the vessel, provides high flexibility, and promotes proper apposition to the vessel wall (9). These design innovations, along with the development of smaller coils, may result in fewer complications and better efficacy when treating tiny aneurysms. Although previous studies have confirmed Atlas stent safety and efficacy (10–12), they included few patients with tiny wide-necked aneurysms. This study aimed to report our preliminary experience with Atlas stent-assisted coiling of tiny wide-necked aneurysms. We also examined safety and efficacy and evaluated risk factors for procedure-related complications.

## Materials and methods

### Patient population and data collection

We performed a retrospective analysis of consecutive adult patients who underwent Atlas stent-assisted coiling of tiny wide-necked intracranial aneurysms between August 2020 and May 2022 in our institution. All aneurysms were diagnosed using digital subtraction angiography (DSA). Tiny was defined as a maximum diameter of ≤ 3 mm. Wide-necked was defined as a dome-to-neck ratio < 2. Patients with ruptured and unruptured saccular aneurysms were included. Those with blood blister-like aneurysms, which have a broad base, lack an identifiable neck, and originate from a nonbranching portion of an artery (13), were excluded. We also excluded patients who did not have postoperative imaging follow-up. The study was approved by the institutional review board of Beijing Tiantan Hospital. All patients provided written informed consent.

The following baseline clinical data were recorded: age, sex, cigarette smoking, alcohol intake, hypertension, diabetes mellitus, and clinical presentation. Imaging data were analyzed to record aneurysm size, shape, location, rupture status, and dome-to-neck ratio. Hunt and Hess's grade was recorded in patients who presented with aneurysmal rupture.

### Endovascular treatment and antiplatelet regimen

Patients with unruptured aneurysms were premedicated with aspirin 100 mg and clopidogrel 75 mg daily for at least 5 days. Patients with ruptured aneurysms received 300 mg loading doses of both aspirin and clopidogrel 4 h before the procedure. All procedures were performed *via* the femoral approach under general anesthesia and full anticoagulation with heparin (targeted activated clotting time was two to three times above the patient's baseline value). A triaxial guide-catheter system using a 6-Fr Cook (Cook Medical, Bloomington, IN, USA) or 6-Fr Neuron MAX (Penumbra, Alameda, California, USA) long sheath, 5-Fr or 6-Fr Navien (Covidien, Irvine, California, USA) intermediate support catheter, and Excelsior SL-10 or XT-17 microcatheter (Stryker Neurovascular) was used to deploy the stent. Aneurysm morphology and parent arterial structure were assessed using a three-dimensional rotational angiography and the proper working projection was selected. An Echelon-10 microcatheter (Medtronic, Dublin, Ireland) was then placed into the aneurysm lumen. An Excelsior SL-10 or XT-17 microcatheter was placed into the parent artery under microguidewire guidance. Aneurysm coiling was performed using the jailing technique; if this failed, the trans-cell technique (through the struts) was performed. All endovascular procedures were performed by neurointerventionalists with more than 10 years of experience. Aspirin 100 mg and clopidogrel 75 mg daily were continued for at least 3 months after the procedure, and then aspirin alone for 6 months or life.

### Postoperative evaluation and follow-up

Procedure-related complications were categorized as hemorrhagic, ischemic, or other. Hemorrhagic complications were defined as visualization of contrast leakage from the aneurysm or ruptured vessel during the procedure or visualization of intracranial hemorrhage on an imaging study performed after the procedure. Ischemic complications were defined as thromboembolic events associated with re-treatment, namely persistent focal neurological deficit, transient ischemic attack, or cerebral infarction.

Clinical outcome was evaluated immediately after the procedure and 3, 6, 12, and 24 months after the procedure *via* outpatient clinic visits or phone calls using the modified Rankin Scale (mRS). Clinical outcome was classified as favorable (mRS score 0–2) or poor (mRS score 3–6). Morbidity was defined as any procedure-related neurological deterioration that caused an increase in the mRS score. Imaging follow-up (DSA or computed tomography angiography) was performed 6, 12, and 24 months after the procedure and was evaluated using the Raymond–Roy (RR) occlusion classification system: grade I, complete occlusion; grade II, residual neck; and grade III, residual aneurysm (14).

### Statistical analysis

Statistical analyses were performed using SPSS software version 24.0 (IBM Corp., Armonk, NY, USA). Continuous variables with a normal distribution are expressed as means with standard deviation (SD); those with a skewed distribution are expressed as medians with an interquartile range. Categorical variables are expressed as numbers with percentages. Continuous variables were compared using the Student's *t*-test or Wilcoxon rank-sum test, as appropriate. Categorical variables were compared using the chi-square or Fisher's exact test as appropriate. Variables with *P* < 0.1 in univariate logistic regression analyses were included in the multivariate analysis to determine independent risk factors for procedure-related complications. *P* < 0.05 was considered significant.

## Results

### Patient and aneurysm characteristics

A total of 46 tiny wide-necked intracranial aneurysms in 46 patients (32 women, 14 men) were included for analysis. The mean patient age was 56.1 years (range, 39–74). A total of 10 patients presented with a ruptured aneurysm. The Hunt-Hess grade was I in five patients, II in three, and III in two. All patients with an unruptured aneurysm had an mRS score < 2 on admission. Of the 46 aneurysms, the location was anterior circulation in 37 (80.4%) and posterior circulation in 9 (19.6%). The mean aneurysm size was 2.5 ± 0.3 mm (range, 1.9–3.0). Mean dome-to-neck ratio was 1.0 ± 0.4 (range, 0.4–2.0). Patient and aneurysm characteristics are shown in Table 1.

**Table 1:** Patient and aneurysm characteristics.

**Characteristics**	**No. (%)**
Total number of patients	46
Age (years) (mean ± SD)	56.1 ± 9.2
Female	32 (69.6)
Comorbidities	
Hypertension	36 (78.3)
Diabetes mellitus	6 (13.0)
Hyperlipidemia	4 (8.7)
Coronary heart disease	5 (10.9)
Smoking	12 (26.1)
Alcohol use	7 (15.2)
Presentation	
Incidental	25 (54.3)
Chronic Headache/dizziness	11 (24.0)
Acute SAH	10 (21.7)
Total number of aneurysms	46
Aneurysm size (mm) (mean ± SD)	2.5 ± 0.3
Aneurysm neck width (mm) (mean ± SD)	2.2 ± 0.4
Dome/neck ratio (mean ± SD)	1.0 ± 0.4
Parent artery diameter proximal (mm) (mean ± SD)	2.6 ± 0.8
Parent artery diameter distal (mm) (mean ± SD)	2.2 ± 0.8
Aneurysm location	
Anterior circulation	37 (80.5)
ICA	7 (15.2)
MCA	6 (13.0)
ACA	6 (13.0)
Anterior choroidal artery	3 (6.5)
AComA	11 (24.0)
PComA	4 (8.8)
Posterior circulation	9 (19.5)
BA	5 (10.8)
PCA	1 (2.2)
PICA	3 (6.5)
Ruptured aneurysm	10 (21.7)
Bifurcation aneurysm	37 (80.4)
Procedure duration (minutes) (mean ± SD)	110.3 ± 31.7

SD, standard deviation; SAH, subarachnoid hemorrhage; ICA, internal carotid artery; MCA, middle cerebral artery; ACA, anterior cerebral artery; AComA, anterior communicating artery; PComA, posterior communicating artery; BA, basilar artery; PCA, posterior cerebral artery; PICA, posterior inferior cerebellar artery.

### Postprocedural angiographic and clinical outcomes

**Figure 1:**
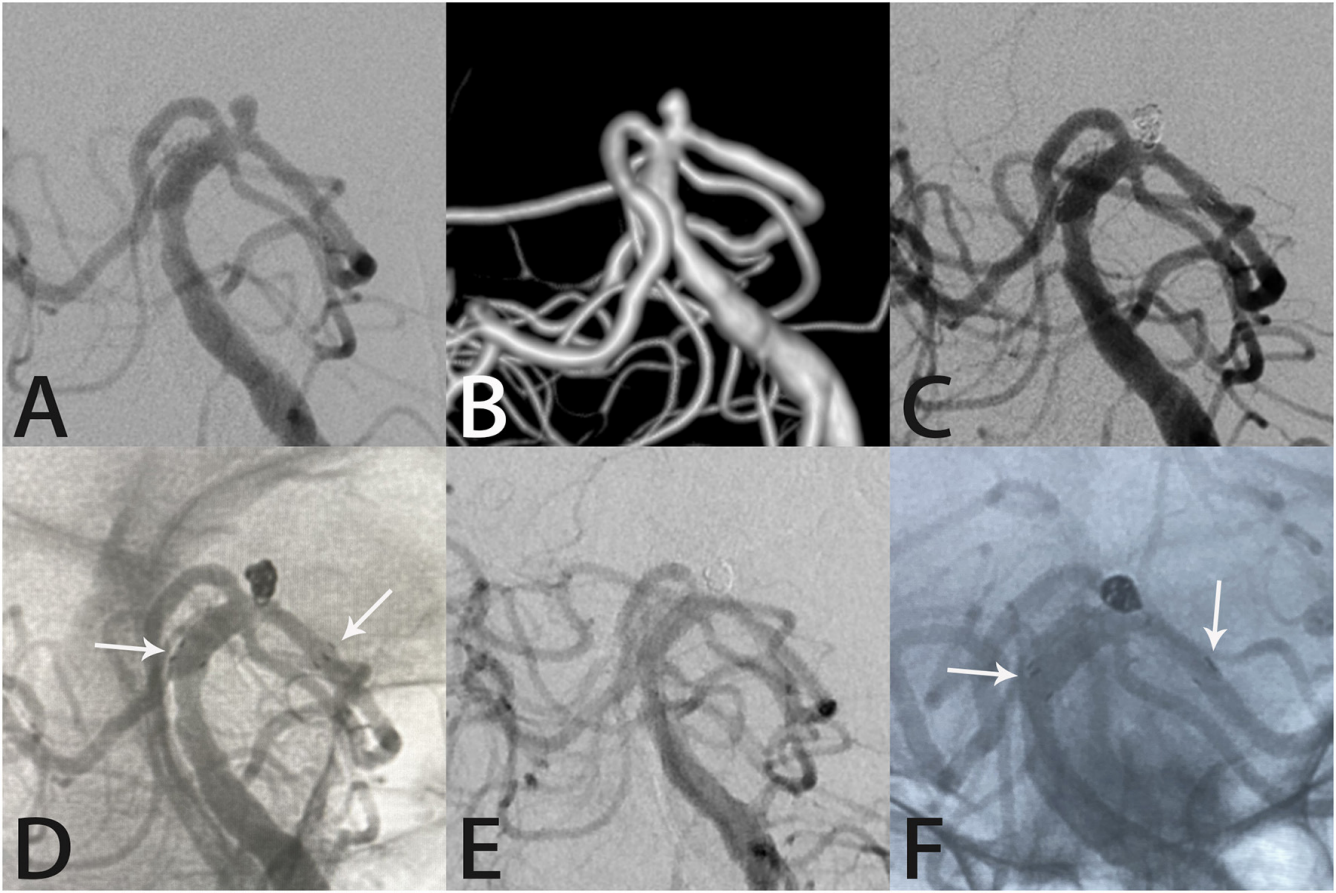
Images from a 66-year-old woman with a tiny left posterior cerebral artery aneurysm. Digital subtraction angiography with three-dimensional reconstruction images demonstrated a left posterior cerebral artery aneurysm **(A,B)**. Angiography immediately after Atlas stent-assisted coiling showed complete aneurysm occlusion **(C)**. An unsubtracted image shows the three radiopaque markers (white arrows) at the proximal and distal ends of the Atlas stent (4.0 × 15 mm) and the coils within the aneurysm sac **(D)**. Follow-up angiography 6 months later showed complete aneurysm occlusion, parent artery patency, stability of the Atlas stent (white arrows indicate the ends of the stent), and the coils densely packed within the aneurysm sac **(E,F)**.

**Figure 2:**
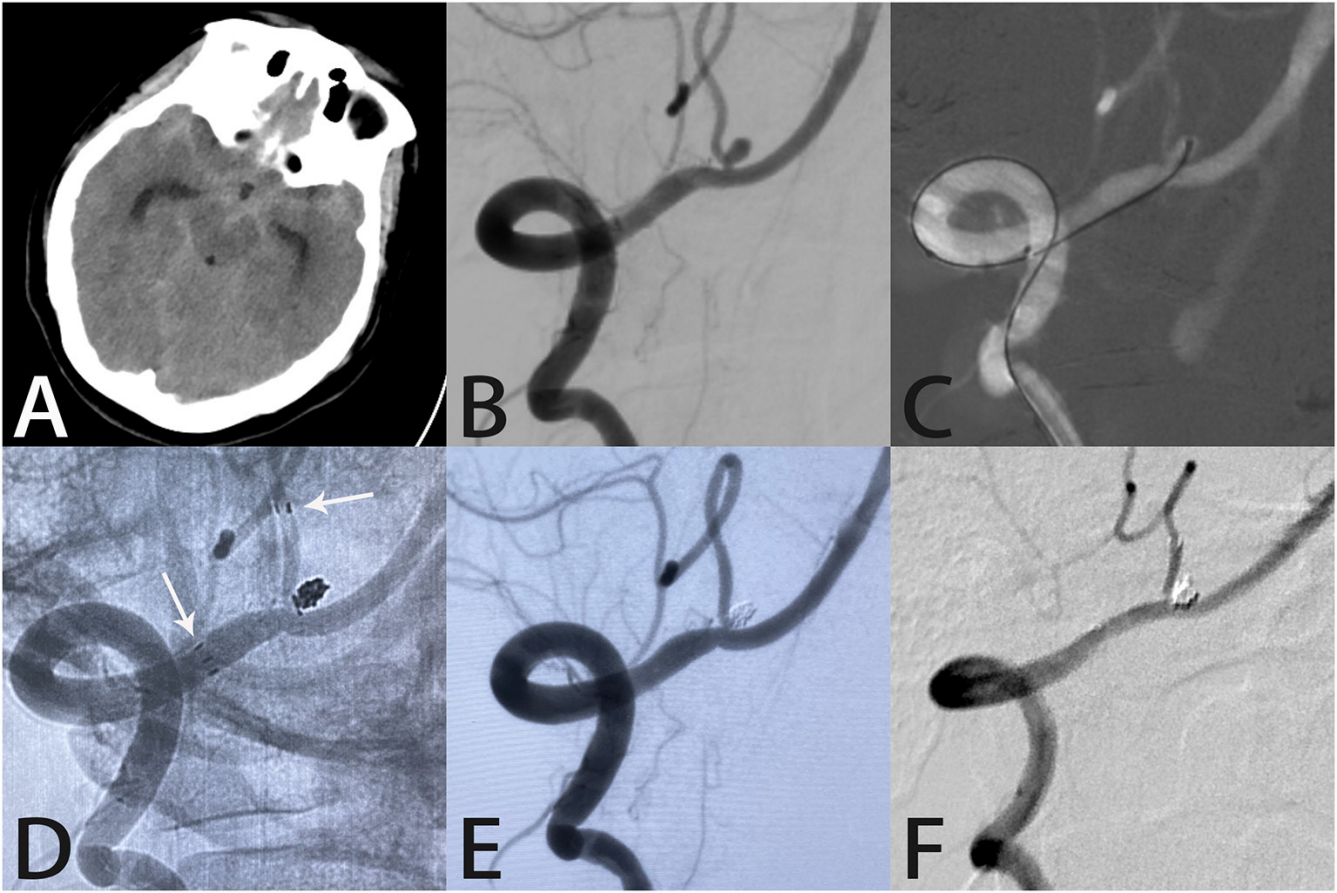
Images from a 45-year-old woman with a tiny ruptured right posterior inferior cerebellar artery aneurysm. Computed tomography showed subarachnoid hemorrhage in the lateral fissure and basal cisterns **(A)**. Angiography demonstrated a right posterior inferior cerebellar artery aneurysm **(B)**. A microcatheter was delivered into the aneurysm sac **(C)**. An unsubtracted image showed the distal end of the Atlas stent (3.0 × 15 mm) was placed in the right posterior inferior cerebellar artery with the proximal end in the right vertebral artery (white arrows indicate the ends of the stent) **(D)**. Angiography immediately after the procedure showed complete aneurysm occlusion and unobstructed blood flow in the posterior inferior cerebellar artery **(E)**. Follow-up angiography 7 months later showed complete aneurysm occlusion with the coils densely packed within the aneurysm sac **(F)**.

Atlas stent deployment was successful in all patients (100% technical success rate). A single stent was placed in 43 cases (93.5%) and two stents were placed in 3 (6.5%). Immediate postprocedural angiography showed complete occlusion (RR grade I) in 38 patients (82.6%), neck remnant (RR grade II) in 7 (15.2%), and partial occlusion (RR grade III) in 1 (2.2%). Complete occlusion was achieved in all 10 patients with a ruptured aneurysm. Figures 1, 2 show representative cases. Postprocedural angiographic and clinical outcomes are shown in Table 2.

**Table 2:** Postprocedural and follow-up angiographic and clinical outcomes.

**Characteristics**	**No. (%)**
Successful stent placement	46 (100)
Number of stents	
Single Atlas	43 (93.5)
Double Atlas	3 (6.5)
Post-procedural immediate aneurysm occlusion, RROC	
I	38 (82.6)
II	7 (15.2)
III	1 (2.2)
Procedure-related complications	
Intraprocedural aneurysm rupture	2 (4.3)
Acute thrombosis	2 (4.3)
Coil migration	1 (2.2)
Angiographic follow-up available	46 (100)
Aneurysm occlusion at follow-up, RROC (*n =* 46)	
I	41 (89.1)
II	5 (10.9)
Follow-up time (months) (mean±SD)	8.4 ± 2.1
Clinical follow-up available	46 (100)
Follow-up mRS scores	
0	259 (70.6)
1	86 (23.4)
2	12 (3.3)
3	1 (2.2)
Follow-up time (months) (mean±SD)	9.7 ± 1.6

RROC, Raymond–Roy occlusion classification; SD, standard deviation; mRS, modified Rankin scale.

**Figure 3:**
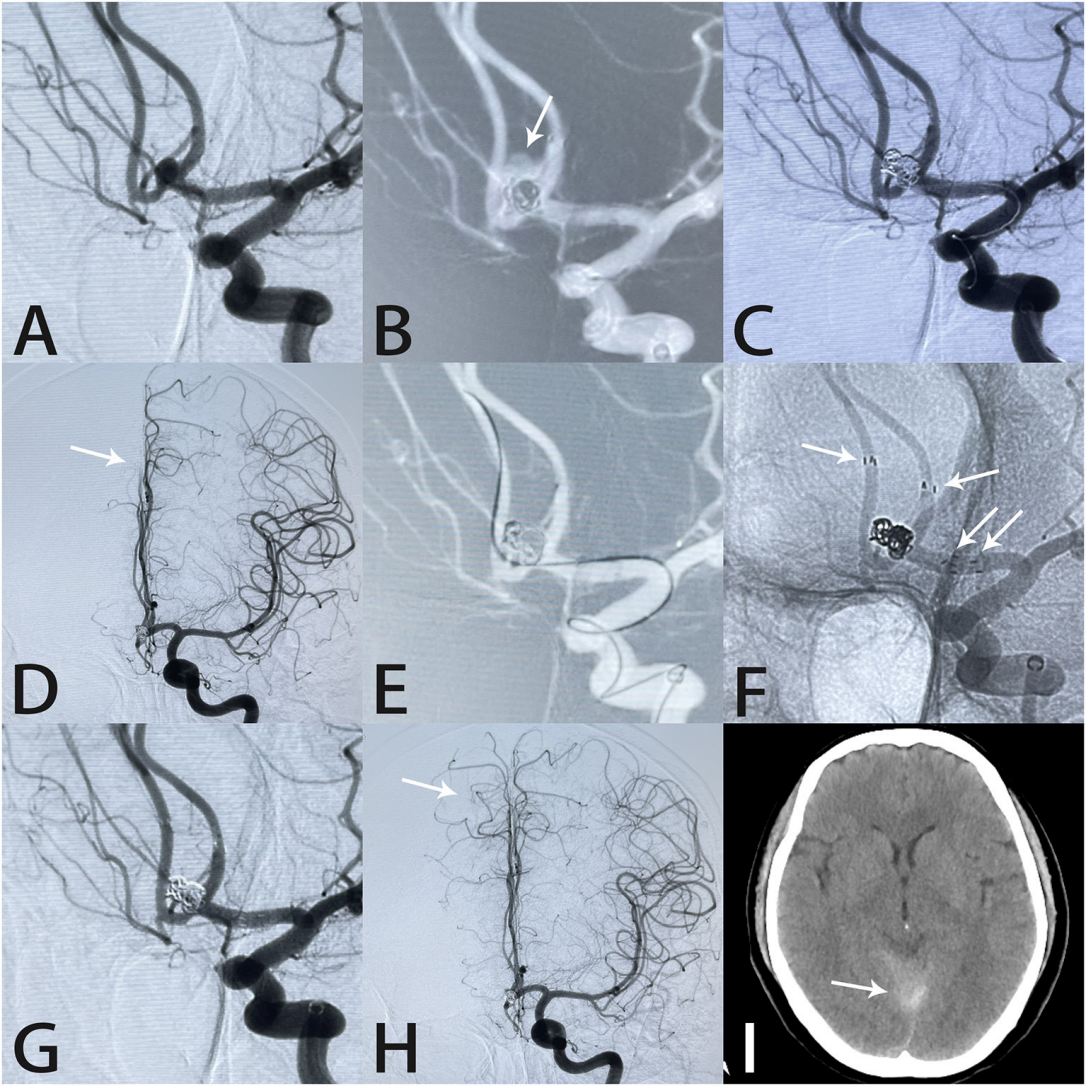
Images from a 56-year-old woman with a tiny anterior communicating artery aneurysm. Angiography demonstrated an anterior communicating artery aneurysm **(A)**. The aneurysm (white arrow) ruptured during coil placement **(B)**. Rapid deployment of additional coils controlled the hemorrhage **(C)**. Intraprocedural angiography showed slow blood flow in the A2 segment of the right anterior cerebral artery (white arrow) **(D)**. An Echelon-10 microcatheter was navigated into the right anterior cerebral artery in preparation for Atlas stent deployment **(E)**. Two Atlas stents formed a “Y-shape” (white arrows) **(F)**. Angiography immediately after the procedure showed complete aneurysm occlusion and unobstructed blood flow in the A2 segment (white arrow) **(G,H)**. Postprocedural computed tomography showed subarachnoid hemorrhage in the tentorium cerebelli **(I)**.

Procedure-related complications occurred in five patients (10.8%), including two intraprocedural aneurysm ruptures, two acute thromboses, and one case of coil migration. No procedure-related death occurred. Both cases of intraprocedural rupture occurred in anterior communicating artery aneurysms. In one, aneurysm perforation occurred during coil placement. Although the rapid deployment of additional coils successfully controlled the hemorrhage, the redundant coils protruded into the parent artery and slowed blood flow in the A2 segment of the right anterior cerebral artery. Therefore, we placed another Atlas stent such that the two stents formed a “Y-shape.” Angiography immediately after the procedure showed complete aneurysm occlusion and unobstructed A2 segment blood flow. The patient experienced transient headaches following the procedure and computed tomography showed only a small amount of SAH (Figure 3). On the other, the patient developed hemiparesis from an intracranial hematoma caused by intraprocedural re-rupture of a ruptured aneurysm; the mRS score was 4 at discharge, which improved to 3 at the last follow-up. Two patients developed in-stent thrombosis during the procedure and were immediately treated with intra-arterial thrombolysis using tirofiban. Recanalization was achieved in both without any clinical sequelae. Coil migration occurred in one patient during coiling, which was treated using Atlas stent deployment to prevent coil protrusion into the parent artery. This patient remained neurologically intact throughout the follow-up. Procedural morbidity and mortality rates were 2.2% and 0, respectively.

### Follow-up angiographic and clinical outcomes

The mean angiographic follow-up was 8.4 ± 2.1 months (range, 6–16). Complete occlusion (RR grade I) was achieved in 41 patients (89.1%) and a neck remnant (RR grade II) was present in 5 (10.9%). No aneurysms recurred and no in-stent stenosis developed during follow-up, even in the three patients treated with the Y-stenting technique.

The mean clinical follow-up was 9.7 ± 1.6 months (range, 8–18). Clinical outcome was favorable in 45 patients (97.8%) and poor in 1 (2.2%). The mRS score was 3 in the single patient with poor clinical outcomes. Follow-up angiographic and clinical outcomes are shown in Table 2.

### Risk factors for procedure-related complications

Univariate logistic regression showed that aneurysm size (odds ratio [OR] 4.538; 95% confidence interval [CI], 1.068–13.489; *P* = 0.045) was significantly associated with procedure-related complications. However, none of the variables examined was an independent risk factor for procedure-related complications in multivariate analysis (Table 3).

**Table 3:** Univariate and multivariate analyses of risk factors for procedure-related complications.

**Parameters**	**Procedure-related complications**		**Univariate analysis**		**Multivariate analysis**	
	**Yes (*n =* 5)**	**No (*n =* 41)**	**OR (95% CI)**	***P*-value**	**OR (95% CI)**	***P*-value**
Age (years)	53.4 ± 10.7	56.4 ± 9.1	0.963 (0.868–1.070)	0.487		
Female, yes	4 (80.0)	28 (68.3)	0.538 (0.055–5.306)	0.596		
Hypertension, yes	3 (60.0)	33 (80.5)	0.364 (0.052–2.553)	0.309		
Current smoker, yes	1 (20.0)	11 (26.8)	0.682 (0.069–6.784)	0.744		
Regular alcohol abuse, yes	1 (20.0)	6 (14.6)	1.458 (0.138–15.387)	0.754		
Ruptured aneurysm, yes	1 (20.0)	9 (22.0)	0.889 (0.088–8.979)	0.920		
Aneurysm size (mm)	2.9 ± 0.1	2.5 ± 0.3	4.538 (1.068–13.489)	0.045	3.868 (0.411–25.781)	0.094
Aneurysm neck width (mm)	2.5 ± 0.3	2.2 ± 0.4	5.672 (0.738–30.518)	0.093	4.436 (0.142–37.723)	0.187
Dome/neck ratio	0.9 ± 0.4	1.0 ± 0.4	0.348 (0.019–6.467)	0.479		
Parent artery diameter proximal (mm)	2.4 ± 0.4	2.7 ± 0.8	0.512 (0.111–2.369)	0.392		
Parent artery diameter distal (mm)	2.0 ± 0.2	2.3 ± 0.8	0.522 (0.127–2.147)	0.368		
Anterior circulation, yes	4 (80.0)	32 (78.0)	1.125 (0.111–11.365)	0.920		
Ruptured aneurysm, yes	1 (20.0)	9 (22.0)	0.889 (0.088–8.979)	0.892		
Procedure duration (minutes)	126.1 ± 20.6	108.4 ± 32.5	1.014 (0.990–.039)	0.250		

OR, odds ratio; CI, confidence interval.

## Discussion

Although guidelines for the treatment of tiny intracranial aneurysms are lacking, commonly applied strategies include surgical clipping and endovascular embolization. Clipping is not ideal for treating tiny aneurysms because it usually causes parent artery narrowing and is associated with a high incidence of complications (15). In a series of 32 tiny aneurysms (≤ 3 mm) that were surgically clipped, Rahmanian et al. reported a 30.8% intraoperative aneurysm rupture rate and 11.5% mortality (16). Considering the high rates of morbidity and mortality associated with neurosurgical clipping, safer and more effective treatment methods are needed for tiny aneurysms, especially those that have a wide neck. Endovascular embolization can achieve permanent occlusion in up to 85% of intracranial aneurysms and has a lower procedure-related hemorrhage rate (17, 18). Since the publication of the International Subarachnoid Aneurysm Trial, the treatment paradigm for intracranial aneurysms has shifted from surgical clipping to endovascular embolization (19). Moreover, recent technological advances, such as smaller coils and smoother microcatheters, and accumulated operator experience have improved the safety and efficacy of endovascular treatment of tiny aneurysms (20). In a meta-analysis that included 1,105 tiny aneurysms (844 ruptured and 261 unruptured), immediate and long-term complete occlusion rates were 85 and 91%, respectively, and rates of intraprocedural rupture and thromboembolism were 7 and 4%, respectively (3).

Since the introduction of the initial Neuroform stent (Stryker Neurovascular) in 2002, various commercially available intracranial stents have followed. The Atlas stent is the successor to the Neuroform stent and has a structure that is compatible with a 0.0165-inch microcatheter, which enables safer and easier treatment of tiny aneurysms on small vessels. Several studies have demonstrated the safety and effectiveness of the Atlas stent for treating both unruptured and ruptured intracranial aneurysms (10, 11). Compared with the LVIS Jr stent (MicroVention, Inc., Aliso Viejo, CA, USA), the Atlas stent is associated with a higher occlusion rate and lower in-stent stenosis rate (21). However, safety and efficacy data regarding Atlas stent-assisted coiling for treating tiny aneurysms is lacking.

Stent assistance during coiling assists with coil placement improves coverage of the aneurysm neck and increases coil packing density. In addition, the presence of stent wires across the aneurysm neck may provide a structural basis for endothelialization and improve hemodynamic conditions. In our series, Atlas stent deployment was successful in all patients, similar to the results of two other studies (22, 23). Table 4 summarizes the results of six studies that examined stent-assisted coil embolization of tiny aneurysms published within the past 10 years. Collectively, the complete occlusion rates immediately after the procedure and at the last follow-up were 64.2 and 87.4%, respectively. In our study, the corresponding rates were 82.6 and 89.1%, respectively. Corresponding rates for the LVIS stent (MicroVention, Inc.) were 40.6 and 82.1%, respectively, while those for conventional stents were 37.8 and 80%, respectively (22, 27). A meta-analysis of small intracranial aneurysms (≤ 3 mm) treated using endovascular techniques reported postoperative and follow-up complete occlusion rates of 85% and 91%, respectively (3).

**Table 4:** Summary of studies examining stent-assisted coil embolization of tiny aneurysms (≤ 3 mm).

**References**	**Stent type**	**No. of aneurysms**	**Rupture/** **Unrupture**	**Mean aneurysm** **size (mm)**	**Immediate complete** **occlusion (%)**	**Complete occlusion** **at the last** ** follow-up (%)**	**Complications (%)**
Zheng et al. (24)	Enterprise/Neuroform/ Solitaire	52	10/42	2.6	86.5	93	4 (8)
Wu et al. (22)	LVIS	32	32/0	2.28	40.6	82.1	1 (3.6)
Li et al. (2)	Enterprise/Solitaire	16	16/0	1.7	56.3	93.8	2 (12.6)
Zhou et al. (20)	LVIS	42	8/34	2.4	76.2	90.5	2 (4.8)
Zhao et al. (25)	Enterprise/Neuroform/ LEO	17	17/0	2.3	58.8	NA	1 (5.9)
Zhang et al. (26)	Solitaire	9	9/0	NA	66.7	77.8	1 (11.1)
Total (mean)		168 (28)	15/13	2.3	64.2	87.4	11 (6.5)

**Figure 4:**
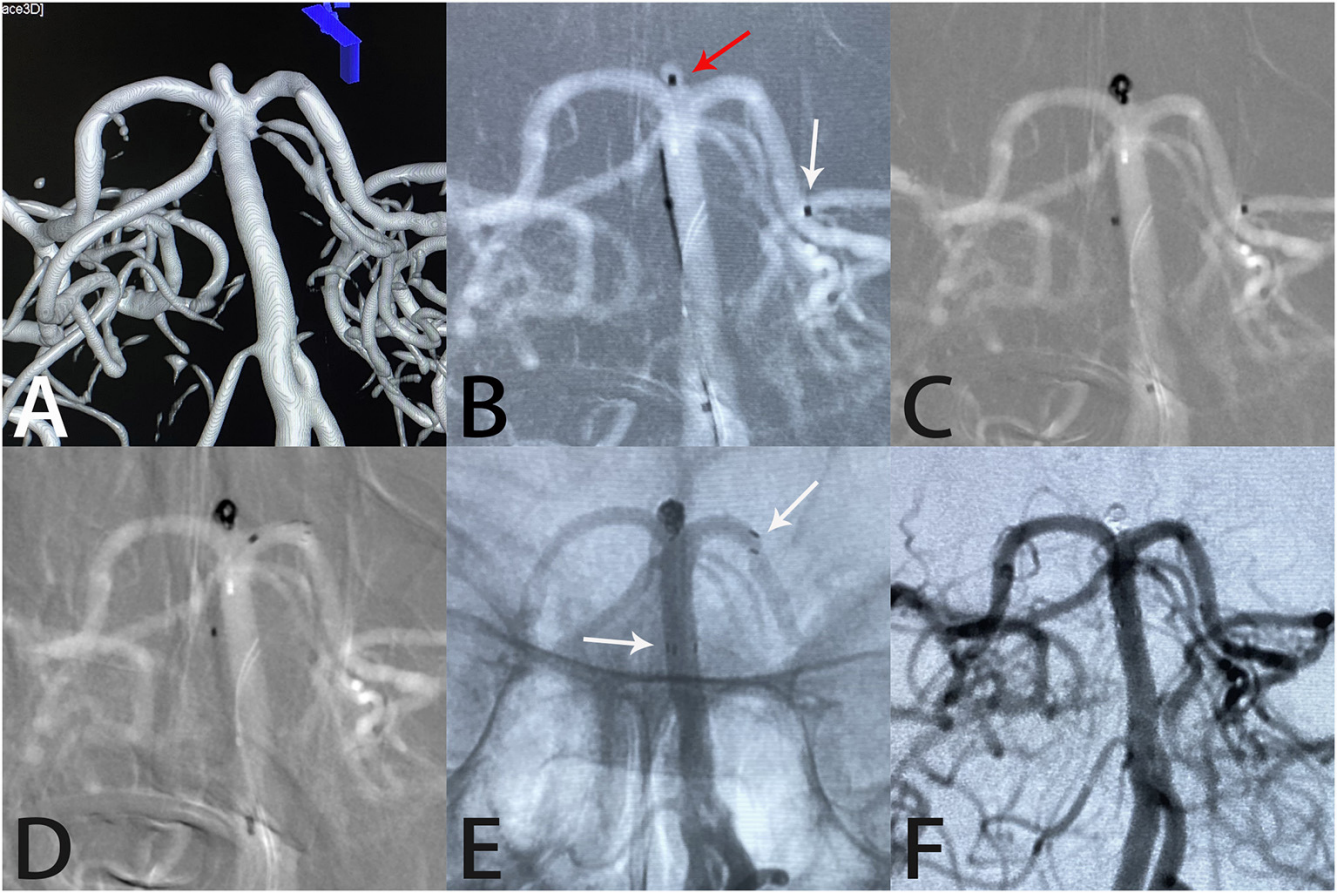
Images from a 65-year-old woman with a tiny basilar artery aneurysm. Three-dimensional digital reconstruction angiography demonstrated a basilar artery aneurysm **(A)**. An Echelon-10 microcatheter was first navigated into the left posterior cerebral artery in preparation for deployment of the Atlas stent (white arrow); then, another Echelon-10 microcatheter was placed into the aneurysm sac (red arrow) for coil placement **(B, C)**. An Atlas stent (3.0 × 15 mm) was deployed through another microcatheter to cover the aneurysm neck **(D)**. An unsubtracted image showed that the coils were packed densely within the sac and the Atlas stent was fitted well against the vessel wall (white arrows indicate the ends of the stent) **(E)**. Angiography immediately after the procedure showed complete aneurysm occlusion and unobstructed blood flow in the left posterior cerebral artery **(F)**.

The Atlas stent may be associated with a higher rate of complete occlusion than other stents because its design allows for a lower profile delivery, improved trackability, and higher conformability to the vessel wall. Available diameters range from 3.0 to 4.5 mm and lengths range between 15 and 30 mm; therefore, the Atlas stent can be used to treat tiny aneurysms located on small distal vessels. Furthermore, dense coil packing was performed in all aneurysms in our study, regardless of rupture status, and the jailing technique was used in most cases. This technique, in which the coil delivery catheter is placed into the aneurysm before stent deployment (Figure 4), may be safer than the trans-cell coiling technique for treating tiny wide-necked aneurysms. With the trans-cell technique, the stent is deployed first and then the coil delivery catheter is delivered into the aneurysm through the stent interstices; however, catheter navigation can be difficult and any unexpected movement may pierce the aneurysm wall and cause hemorrhage.

Intraprocedural rupture during endovascular aneurysm treatment usually leads to poor clinical outcomes. Several studies have shown that the risk of intraprocedural aneurysm rupture appears to be higher for aneurysms < 3 mm in diameter (28, 29). In addition, a 2016 meta-analysis of aneurysms < 3 mm in diameter reported a 7% intraprocedural rupture rate (3), which is higher than the 4.3% rate in our series. However, we excluded patients with blood blister-like aneurysms, which have a higher risk of intraprocedural rupture.

Intraprocedural rupture prevention is critical for success when treating tiny aneurysms. We prefer to use coils that are ultra-soft and slightly smaller than the maximum diameter of the aneurysm, which enables a high packing density. In addition, we place the microwire tip near the neck of the aneurysm rather than inside it to avoid piercing the aneurysm. Furthermore, to achieve adequate maneuverability, the shape of the tip should be adjusted based on the angle between the parent artery and the aneurysm. Delicate maneuvering contributes to lowering the risk of intraprocedural rupture. Li et al. (2) developed the stent-assisted coil-jailing technique, which staples the redundant coil tail between the stent and the parent artery wall, which lowers the risk of intraprocedural rupture and coil displacement.

The Atlas stent may be associated with an increased risk of intraprocedural thrombosis. Intraprocedural thrombosis is mainly associated with poor stent–vessel wall apposition, inadequate antiplatelet therapy, longer procedure time, and thrombogenicity of endovascular embolization materials (30, 31). In our study, in-stent thrombosis occurred in two patients (4.3%), which is slightly higher than the rate reported by Ioanniaid et al. (3.1%) (32). Fortunately, the tirofiban administration resulted in recanalization in both.

Our overall complication rate of 10.8% was higher than the collective rate compiled from our literature review (6.5%). Although aneurysm size was associated with procedure-related complications in our univariate analysis, the multivariate analysis did not identify any independent risk factors.

Most neurointerventionalists are reluctant to place stents in patients with a ruptured aneurysm because they are in a hypercoagulable state and at risk for in-stent thrombosis and associated ischemic complications. In addition, the administration of antiplatelet drugs may cause intracranial hemorrhage again. However, we found no significant difference in procedure-related complications between ruptured and unruptured aneurysms.

### Limitations

Our study has several limitations. First, it was retrospective in design and was conducted in a single center. Therefore, selection bias may have been introduced. Second, the mean angiographic follow-up was only 8.4 months, which is too short to determine the true final complete occlusion rate. Third, we did not compare the results of stent-assisted coiling and coiling alone. This comparison would better demonstrate the effect of stenting on outcomes in endovascular treatment of tiny aneurysms.

## Conclusion

Atlas stent-assisted coiling of tiny wide-necked intracranial aneurysms is safe and effective. Outcomes and occlusion rates are favorable and morbidity is low; however, the complication rate may be higher in larger tiny aneurysms. Prospective multicenter studies with long-term follow-ups are warranted.

## Data availability statement

The raw data supporting the conclusions of this article will be made available by the authors, without undue reservation.

## Ethics statement

This study was reviewed and approved by the Ethics Committee of Beijing Tiantan Hospital. Written informed consent to participate in this study was provided by the patients or their legal guardian/next of kin.

## Author contributions

LD and XC collected the clinical data, performed the statistical analysis, and wrote the manuscript. LZ, ZZ, and QP helped to collect the clinical data. PL and ML helped revise the manuscript, designed the research, and handled funding and supervision. ML approved the final version on behalf of all authors. All authors read and approved the final manuscript.

## Funding

This study was supported by the Youth Program of National Natural Science Foundation of China (Grant no. 81901197).

## Conflict of interest

The authors declare that the research was conducted in the absence of any commercial or financial relationships that could be construed as a potential conflict of interest.

## Publisher's note

All claims expressed in this article are solely those of the authors and do not necessarily represent those of their affiliated organizations, or those of the publisher, the editors and the reviewers. Any product that may be evaluated in this article, or claim that may be made by its manufacturer, is not guaranteed or endorsed by the publisher.
